# Role of Fibulins in Embryonic Stage Development and Their Involvement in Various Diseases

**DOI:** 10.3390/biom11050685

**Published:** 2021-05-02

**Authors:** Deviyani Mahajan, Sudhakar Kancharla, Prachetha Kolli, Amarish Kumar Sharma, Sanjeev Singh, Sudarshan Kumar, Ashok Kumar Mohanty, Manoj Kumar Jena

**Affiliations:** 1 Department of Biotechnology, School of Bioengineering and Biosciences, Lovely Professional University, Phagwara, Punjab 144411, India; deviyanimahajan.123@gmail.com (D.M.); amarish.19824@lpu.co.in (A.K.S.); sanjeev.15935@lpu.co.in (S.S.); 2 Devansh Lab Werks, 234 Aquarius Drive, Homewood, AL 35209, USA; sudhakar@devlabwerks.com; 3 Microgen Health Inc., 14225 Sullyfield Cir Suite E, Chantilly, VA 20151, USA; Prachetha@microgenhealth.com; 4 Animal Biotechnology Centre, National Dairy Research Institute, Karnal, Haryana 132001, India; kumarsudershan@gmail.com (S.K.); ashokmohanty1@gmail.com (A.K.M.)

**Keywords:** fibulins, embryonic stage, cancer, heritable disorders

## Abstract

The extracellular matrix (ECM) plays an important role in the evolution of early metazoans, as it provides structural and biochemical support to the surrounding cells through the cell–cell and cell–matrix interactions. In multi-cellular organisms, ECM plays a pivotal role in the differentiation of tissues and in the development of organs. Fibulins are ECM glycoproteins, found in a variety of tissues associated with basement membranes, elastic fibers, proteoglycan aggregates, and fibronectin microfibrils. The expression profile of fibulins reveals their role in various developmental processes such as elastogenesis, development of organs during the embryonic stage, tissue remodeling, maintenance of the structural integrity of basement membrane, and elastic fibers, as well as other cellular processes. Apart from this, fibulins are also involved in the progression of human diseases such as cancer, cardiac diseases, congenital disorders, and chronic fibrotic disorders. Different isoforms of fibulins show a dual role of tumor-suppressive and tumor-promoting activities, depending on the cell type and cellular microenvironment in the body. Knockout animal models have provided deep insight into their role in development and diseases. The present review covers details of the structural and expression patterns, along with the role of fibulins in embryonic development and disease progression, with more emphasis on their involvement in the modulation of cancer diseases.

## 1. Introduction

The extracellular matrix (ECM) is the non-cellular component that provides structural scaffolding to the surrounding cells. It also provides the critical biochemical support required for intracellular and cell–cell communication, differentiation, and homeostasis. ECM is highly diverse with respect to its composition and function. It plays a pivotal role in tissue differentiation, the development of organs during the embryonic stage, and modulation in the bioavailability of growth factors in multi-cellular organisms. The two main classes of extracellular macromolecules are glycosaminoglycans (GAGs) and fibrous proteins, which constitute the matrix. GAGs usually occupy a large space and form covalent bonds with the proteins (forming the proteoglycans), whereas fibrous proteins have adhesive and structural functions. Prominent examples of fibrous proteins include fibulin, fibronectin, collagen, laminin, and elastin. Fibulin proteins are part of a family of eight members, expressed in a variety of tissues and secreted in the ECM as glycoproteins [1].

**Figure 1:**
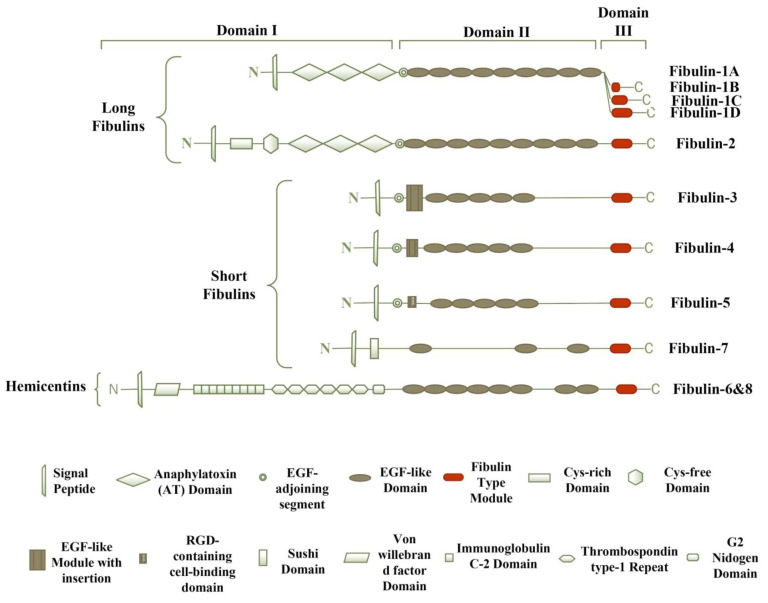
The modular structure of the fibulin family. The eight members of the fibulin family have a similar arrangement and consist of three modules, grouped as domain-I, -II, and -III. Fibulin-5 is the only member of the fibulin family that displays evolutionarily conserved arginine–glycine–aspartic acid (RGD) sequence, which promotes binding to the cell surface integrin receptors. Hemicentins display a unique von Willebrand factor domain.

Fibulins share a common basic structure (Figure 1), and consist of three domains—domains-I, -II, and -III. Domain-I represents the N-terminus, which shows variability among each member of the fibulin family. Domain-II is the central portion of the protein structure, characterized by the variable number of EGF-like modules with calcium-binding sequences (cbEGF). Domain-III is also known as the fibulin type module, which is the unique C-terminus domain with 120–140 amino acids [2]. The expression profile of the fibulin family reveals a prominent expression of fibulins in the areas undergoing epithelial–mesenchymal transition (EMT) during embryonic development [3]. Studies have revealed that fibulins play a vital role during embryonic development in tissue remodeling, basement membrane and elastic fiber structural integrity, and other cellular processes [2,3,4]. These proteins also have a role in wound healing, and are involved in diseases like cancer, Malattia Leventinese, Doyne honeycomb retinal dystrophy, Sjogren’s syndrome, chronic fibrotic disorders, and congenital defects [4].

The present review thoroughly discusses the structural and functional properties of the different isoforms of fibulins, with more focus on their role in development and disease progression. This study will help researchers understand various aspects of fibulin proteins and design their experiments in this area towards a targeted therapeutic approach against various diseases.

## 2. The Fibulin Family 

Based on the length and structure of modules, fibulins have two subgroups. The first subgroup is of long fibulins, which consists of fibulin-1 and fibulin-2, and have a tendency to form dimers [4]. They are larger in size because of the presence of three anaphylatoxin modules and additional EGF-like modules in domains-I and -II, respectively [4]. Reportedly, fibulin-1 has four variants, i.e., A, B, C, and D. Variants A and B are found only in humans, whereas variants C and D are found in zebrafish, mice, nematodes, and chickens. All of these variants show the structural difference in domain-III, i.e., the fibulin-type module. In variant A, domain-III is absent completely; a small portion of domain-III is present in the B variant compared with that of variants C and D [3,5,6]. The main function of fibulin-1 is to mediate cell signal transduction events by binding to other components of ECM, including fibronectin, laminin-1, and versican [3]. In both humans and mice, fibulin-2 exists as a dimer, with each monomeric unit having a molecular weight of ~195-kDa connected by disulfide covalent bond, and plays a vital role in tissue development and remodeling [4]. Fibulin-2 has two extra cysteine-rich modules at the N-terminal end [3].

The second subgroup contains short fibulins, i.e., fibulin-3, -4, -5, and -7, which exist as monomers [7]. Short fibulins are different from the long fibulins in domain-I, as the domain-I short fibulins contain a cbEGF-like module, whereas the domain-I long fibulins contain three anaphylatoxin modules [2]. They play multiple roles in the formation of tissue and its remodeling. In humans, fibulin-3 consists of five splice variants, with a complete or partial absence of domain-I [8]. Fibulin-5 is the only member of short fibulins with an arginine–glycine–aspartic acid (RGD) motif at the N-terminal end, and it promotes binding to the cell surface integrin receptors, as studied in mice [9]. The molecular structure of fibulin-7 in humans has revealed the presence of a central flank containing cbEGF-like repeats and a unique Sushi domain at the N-terminal end, which regulates the complement system and blood coagulation [3,10].

Hemicentins are ECM glycoproteins, identified in the nematode *Caenorhabditis elegans* as evolutionarily conserved ECM proteins, and play important role in the organization of tissues, basement membrane invasion, cell migration, and cell–cell and cell–matrix contacts mainly in the epithelial tissues [11]. The molecular structure of hemicentin-1 and -2 resembles typical fibulin modules; hence, the are classified as fibulin-6 and fibulin-8, respectively [12]. Fibulin-6 and fibulin-8 are characterized by the von Willebrand domains at the amino-terminal end, preceded by tandem repeats of immunoglobulin domains [13].

Signaling molecule transforming growth factor-β (TGF-β) is a key regulator of fibulins, like those of other ECM proteins. It has been observed that the addition of TGF-β to mouse cardiac fibroblast cells up-regulates the fibulin-2 expression and enhances TGF-β signaling [14]. In murine 3T3-L1 fibroblasts, TGF-β induces fibulin-5 transcription and translation through the Smad3 independent pathway, and activates the ERK1/ERK2 and p38 mitogen-activated protein kinase (MAPK) pathway [15]. In human lung fibroblasts, TGF-β stimulates fibulin-5 transcription via the PI3K/AKT pathway [16]. Similarly, in pancreatic ductal adenocarcinoma, an increased expression of fibulin-5 was reported to be induced by TGF-β via the PI3K/AKT signaling pathway [17]. Additionally, it has been observed that in breast and ovarian cancer, estrogen regulates the expression of fibulin-1 by interacting with estrogen receptors [18]. Furthermore, integrin α3β1 plays a pivotal role in regulating the expression of fibulin-2 in the transformed keratinocytes [19]. 

## 3. Role of Fibulins in Embryonic Development

### 3.1. Long Fibulins

#### 3.1.1. Fibulin-1

Fibulin-1 is also known as the BM-90 protein and it is the first member of the fibulin family with a molecular size of 90–100 kDa (Table 1) [20]. The expression of fibulin-1 is observed in the basement membranes, connective tissues, and matrix fibers [21,22]. In avian embryos, the early expression of fibulin-1 has been spotted during the first 23 h of development in the embryonic midline and in the elastin fibers; surrounding structures of the midline; and in the areas undergoing EMT, including the developing myotomes, neural crest, and endocardial cushions [23,24]. In chicken embryos, a higher expression of fibulin-1 has been found in the endocardial cushion of the heart during the development of the cardiac valve and septa in cushion [25]. In humans, it has been reported that fibulin-1 plays an important role by providing elasticity in the connective tissues, along with its involvement in the process of fibrogenesis [21]. Additionally, fibulin-1 is also expressed in the areas of the developing perichondrium, endocardium, endocardial cushion, epicardium, basement membrane of the endothelium, perivascular mesenchyme, basement membrane of skin, periderm, basal layer, mesothelium, basement membrane of the neuroepithelium, and leptomeningeal cell anlagen during the earlier gestational weeks, and in the myocardium, developing tunica adventitia, subepithelial layer, and perineurium during the mid-gestational weeks. The expression of fibulin-1 in the developing mouse embryo remains high in the mesenchymal cells of developing organs such as the kidney, lung, and intestine, and in the cartilage during chondrogenesis. Furthermore, fibulin-1 is expressed highly in parts such as the tongue, intestine, and connective tissues, and in some parts of the central and peripheral nervous system [26]. In humans, an increased expression of fibulin-1 has been observed in the endometrial stromal cells treated with 6α-methyl-17α-hydroxy-progesterone acetate (MPA), suggesting that fibulin-1 plays a vital role in promoting progesterone-mediated actions. MPA is a synthetic progestogen that seems to stimulate fibulin-1 mRNA expression in a dose-dependent manner [27]. During the development of the cardiac outflow tract, fibulin-1 is expressed at the endothelial mesenchymal transition area, and averts the hypercellularity of the proximal endocardial cushion by conquering the outflow tract transition and the aggregation of blood cells [28]. Fibulin-1 interacts with fibroblast growth factor 8 during the development of the embryo, and maintains the neural crest cells (NCCs) and embryo survival [29]. The in vitro study on the expression analysis of fibulin-1 and fibulin-2 in the human corneal fibroblast cells using microarray revealed the expression of both of these two genes along with their binding partners, such as fibronectin, nidogen-1, aggrecan, fibrilin-1, endostatin, laminin alpha-2 chain, and ADAMTS-1 (a disintegrin and metalloproteinase with thrombospondin motif 1) metalloprotease [30]. Further experiments in this study showed the involvement of fibulin-1 in cell motility. This study on the expression pattern of fibulins and their binding partners in corneal fibroblast cells revealed their involvement in the organization of the supramolecular structures in the ECM. The binding of fibulin-1 to nidogen was found to depend on the C-terminal globular domain and the array of EGF-like modules on domain-II of fibulin [31]. 

It was revealed in the mouse embryo study that fibulin-1 deficient mice show a spectrum of disorders, including malformation of cranial nerves, thymic hypoplasia, thinning of the wall of cardiac ventricles, aortic arch artery anomalies, and defects of the ventricular septae. The development of all of these tissues is dependent on the neural crest cells, and fibulin-1 plays a crucial role in the migration and survival of neural crest cells. Hence, fibulin-1 is an important protein required for the development of neural crest derived structures [32]. This protein also plays a role during the morphogenesis of the ventricle by promoting the ADAMTS-1 mediated cleavage of versican, which in turn represses the proliferation of trabecular myocytes [33]. In the kidney of developing mouse embryos, it has been seen that fibulin-1 regulates the proteolysis of proteoglycans, mediated by ADAMTS-1, which shows its role in the morphogenesis of kidneys [34]. Fibulin-1 null mice tissues manifested hemorrhagic conditions during the early developing period, leading to the death of almost all of the mice before birth, because of the malformation of organs [35].

#### 3.1.2. Fibulin-2

For the first time, fibulin-2 was identified in the mouse fibroblast cDNA clones [36]. The expression pattern of this protein partially overlaps with that of fibulin-1, but it has a more restricted expression level than that of fibulin-1 [7]. This protein plays an important role in matrix remodeling, cell migration, and elastogenesis [37]. It interacts with other molecules of ECM, such as tropoelastin, versican, fibronectin, laminin, fibulin-1, fibrillin-1, and nidogen [38]. The interaction of fibulin-2 with tropoelastin during vascular system development serves as a link between the elastin core and fibrillin microfibrils [39]. Fibulin-2 is expressed highly in the endocardial cushion tissue of the developing heart in order to maintain the tensile strength of the cardiac valves [40]. In the early mouse embryonic stage, fibulin-2 is co-expressed with the versican proteoglycan in myoepithelial cells, and allows epithelial budding and the outgrowth of the developing mammary ducts [41]. Its expression is also up-regulated in the developing cardiac valves and the aortic arch vessels during the migration process of transformed mesenchymal cells to the ECM [42]. During organogenesis, its expression remains high in the areas of developing cartilage, including regions such as the larynx, nasal septum, thyroid, and mandible. Furthermore, fibulin-2 is also expressed during smooth muscle development, perichondrium formation (at the time of femur cartilage and vertebral development), and in the region of boundary formation between the individual tissues [26]. In chick embryos, fibulin-2 is expressed at the posterior half sclerotomes, responsible for the shaping of spinal nerves and increasing the repulsive activity of the Sema3A axon. Furthermore, it is also highly expressed in the region of the perinotochord (released from the notochord itself), in order to organize the dorsal root ganglion (DRG) axon projections during development. Additionally, a higher expression of fibulin-2 was spotted in the astrocytes of adult injured mice [43]. In human embryonic development, it is expressed in developing the perichondrium, endocardium, endocardial cushion, epicardium, basement membrane of the endothelium, perivascular mesenchyme, basement membrane of skin, periderm, basal layer, mesothelium, neuroepithelial cells, basement membrane of the neuroepithelium, and spinal nerves during early gestational weeks, and in the tunica adventitia, sub-epithelial layer, and developing ganglia during the mid-gestational weeks [44]. Its expression is up-regulated in wound healing, which justifies its role in the remodeling of tissue [45]. Fibulin-2 acts as a marker to differentiate rat liver fibroblasts from other fibrogenic cells of the liver [46]. Its expression is also found to be higher in solar elastosis, suggesting its involvement in the formation of elastic fiber and microfibrils [47].

Fibulin-2 knockout mice do not develop any anatomical or other evident abnormalities, and they grow normally with normal fertility. The compensatory up-regulation of fibulin-1 has been noticed in aortic and skin tissues [48]. Newborn fibulin-2 knockout mice develop partial abnormalities in the formation of the basement membrane of the skin. These knockout mice show a resemblance to the integrin α3β1 knockout mice with a reduced fibulin-2 induction, suggesting that fibulin-2 is an important protein to induce the stability of the basement membrane [49].

### 3.2. Short Fibulins

#### 3.2.1. Fibulin-3

The glycoprotein fibulin-3 is also known as S15 or T16 or EFEMP1 (EGF-containing fibulin-like extracellular matrix protein 1) protein, and, for the first time, it has been observed in senescent human fibroblasts, established from a patient with Werner syndrome with premature aging [8]. In human tissues, the expression of fibulin-3 is reported to be up-regulated along with the fibulin-4 in the heart, placenta, lungs, and skeletal muscle [2]. In mouse tissues, its expression is high in the lungs, but moderate in the esophagus and low in the aorta. Its expression pattern partially overlaps with the expression pattern of fibulin-1 and fibulin-4 [2,7]. In developing mouse embryos, fibulin-3 expression is highly up-regulated in condensing mesenchyme, and developing cartilage and bone, suggesting its role in the shaping of the skeletal elements in the body [50]. Fibulin-3 expression has been seen in the eyes, lungs, brain, kidney, and heart of adult mice, as well as in the endothelial and epithelial cells throughout the body [7,51].

Fibulin-3 null mice have been found to develop premature aging phenotypes such as decreased body mass and bone density, reduced hair growth and reproductive behavior, and spine deformity, but do not show any macular degeneration. There was reduced elastic fiber formation in the fascia, suggesting that fibulin-3 plays an important role in maintaining the elastic fiber density of fascia [52]. The knocked-in mouse with a R345W missense mutation in the *FIBL3* gene showed macular degeneration [53].

#### 3.2.2. Fibulin-4

The glycoprotein fibulin-4 (also known as EFEMP2 (EGF-containing fibulin-like extracellular matrix protein 2), MBP1 (Mutant p53-Binding Protein 1), or H411) has a molecular weight of ~60 kDa, and it was identified as a paralog of fibulin-3 in EST clones [2,54]. The expression of fibulin-4 has been observed in various tissues throughout the body, with a higher level in the vasculature [2]. This protein plays a pivotal role in activating the enzyme lysyl oxidase (this enzyme catalyzes the collagen and elastin covalent cross-linkage). Fibulin-4 helps to transport the copper ions to lysyl oxidase from ATP7A, in order to form lysine tyrosyl quinine (a cofactor that regulates the function of lysyl oxidase). Hence, this protein is involved in the regulation of the lysyl oxidase enzyme activity and assembly of the ECM [55]. Its expression level is weak in the pancreas, brain, kidneys, lungs, and placenta; moderate in the skeletal muscles; and strong in the heart muscles [3,56]. The expression of fibulin-4 has been revealed in the chondrocytes of the articular region and in the cultured chondrocytes [57]. In large blood vessels, its expression becomes intense towards the adventitia in the outer medial layer [58].

In fibulin-4 deficient mice, the aggregation and development of irregular elastic fibers have been observed, suggesting that fibulin-4 has an indispensable role in elastic fiber development [59]. Mice with a low expression of fibulin-4 showed abnormalities such as the development of aneurysm, abnormalities in the cardiac system, and tears within the aortic wall, suggesting its role in the development of the heart [60]. It has been observed that the degree of aortic aneurysm is inversely proportional to the amount of fibulin-4 in the tissues [61]. Fibulin-4 knocked-out mice are found to be unable to form elastic fibers because of the downregulation of the tropoelastin expression in fibroblast cells. Hence, fibulin-4 plays a vital role in the formation of elastic fibers in fibroblast cells by regulating the expression of tropoelastin [62]. Targeted disruption of fibulin-4 in mice resulted in perinatal lethality in association with hemorrhage due to the rupture of the aorta and the diaphragm, together with the aneurysmal aortic vessels and the emphysematous lungs [59]. The reduced expression of fibulin-4 led to the formation of aneurysm, cardiac abnormality, and dissection of aortic walls. Additionally, homozygous mice with a reduced fibulin-4 expression culminated in the disorganized network of elastic fibers and disturbed the TGF-β signaling pathway [56]. The reduced expression of fibulin-4 in adult mice with the fibulin-4^-/R^ and fibulin-4^R/R^ genotypes developed the features of cystic media degeneration, and there was a smaller number of smooth muscle cells [63]. It seems that fibulin-4 is crucial for the assembly of the elastin fiber in the large conduit and ascending aorta, but not in the muscular/resistant arteries, and elastic fiber assembly has different requirements depending on the types of vessels [64].

#### 3.2.3. Fibulin-5

The glycoprotein fibulin-5 (also known as DANCE (developmental arteries and neural crest EGF-like protein), EVEC (embryonic vasculature epidermal growth factor-like repeat-containing protein), or UP50 (Urine p50 protein) protein) has a molecular weight of ~65 kDa, and plays a vital role in tissue remodeling and vascular system development [65]. This protein was first isolated from the embryonic heart library using the subtraction hybridization technique to identify the gene that regulates the modulation of quiescent vascular smooth muscle cells to a primitive proliferative state [66]. In adult humans, a strong expression of this protein has been observed in the colon, heart, and ovary, whereas the growing mouse showed its expression in maternal endothelial cells, mesenchymal tissue, pericardium, endocardial cushion tissue, and some neural crest cell-derived tissues. Fibulin-5 is also highly expressed in balloon-injured vessels of rats, suggesting its involvement in the remodeling and development of the vascular system [65]. Fibulin-5 expression is up-regulated in endometrial decidual cells during the first trimester to regulate the invasion of extravillous trophoblast cells and the placentation process [67]. In the developing bovine fetal ovary, the expression of this protein is increased during the formation of surface epithelium and tunica albuginea, suggesting its role in ovary development [68]. A strong expression of fibulin-5 was found in rat lungs between the 18th embryonic day and 17th postnatal day, and an in situ hybridization technique revealed its expression in interstitial cells and pulmonary vessels [69]. The presence of fibulin-5 in elastic fiber-enriched tissues indicates its involvement in the formation of elastogenic tissues [9].

The fibulin-5 protein regulates the morphogenesis of the craniofacial skeletal and facial suture of the mice, as fibulin-5 null mice showed postnatal facial defects such as elongation of premaxillary bone, proliferative defects of premaxillo-maxillary suture (PMMS) cells, and defects in the differentiation of PMMS cells into osteoblasts [70]. Additionally, fibulin-5 deficient mice showed symptoms of human cutis laxa syndrome, such as loose skin, emphysematous lungs, and elastic fiber disorganization, revealing its involvement in elastic fiber organization [71]. An electron microscopic examination of fibulin-5 knocked-out mouse tissues showed elastin globules adjacent to the microfibrils and a lower level of matured cross-linked elastin in the dermis, supporting its role in the incorporation of elastin fibers in the bundles [72]. Furthermore, fibulin-5 null mice with vascular injury induced vascular remodeling and displayed a loss of structural integrity and forms severe neointima, revealing the role of this protein in inhibiting the proliferation and migration of smooth muscle cells [73]. This protein has multiple binding sites for other ECM proteins, including fibrillin-1, extracellular superoxide dismutase, latent TGF-β binding protein-2, latent TGF-β binding protein-3, and lysyl oxidase-like protein-1, revealing its role in the formation of the microfibrillar scaffold, elastic fiber assembly, signal transduction, deposition of tropoelastin, and cell–matrix interaction [74,75,76,77,78]. Fibulin-5 acts as an antagonist of vascular endothelial growth factor (VEGF) and inhibits VEGF signaling, resulting in the inhibition of angiogenesis and endothelial cell activities [79].

#### 3.2.4. Fibulin-7

The glycoprotein fibulin-7 (also known as TM14) is the newly introduced member of the fibulin family, with a molecular weight of ~48 kDa. This is an adhesion protein that is expressed in developing teeth; cartilage; placenta; hair follicles; and, more precisely, in preodontoblasts and odontoblasts during molar and incisor development, articular cartilage, and spongio-trophoblasts of the placenta [10]. Fibulin-7 expression is also detected in the endothelial cells of blood vessels and choroid of the eye [80]. In newborn mice, a low expression of this protein has been observed in the kidney. In contrast, in adult mice, a higher expression has been found in the renal tubular epithelium, Bowman’s capsule epithelium, and perivascular regions of the kidney [81].

Fibulin-7 knocked-out mice are healthy and fertile, and do not display any abnormalities related to elastic fiber development [82]. In 2q13 deletion syndrome, fibulin-7 was identified as one of the responsible genes, as the knockdown of fibulin-7 in zebrafish developed the same phenotype as 2q13 deletion syndrome [83]. This protein promotes endothelial cell adhesion and inhibits the formation of the endothelial tube via β1-integrin and heparin sulfate receptors, revealing its role as an angiogenesis inhibitor [84]. It acts as a potential immune-modulator in the inflammatory diseases, as its C-terminal fragment negatively regulates monocyte and macrophage migration, differentiation, and cytokine production [85].

### 3.3. Hemicentins

#### 3.3.1. Fibulin-6

The glycoprotein fibulin-6 is also known as hemicentin-1; has a molecular weight of 600 kDa; and it is expressed in skin fibroblasts, retinal pigment epithelial cells, and retinal endothelial cells [86]. The function of fibulin-6 has been identified in the nematode *C. elegans* and it plays a vital role in the fusion of two basement membranes, as it controls basement membrane linkage by forming punctuate accumulation under the anchor cells [87]. Anchor cells are the key organizers of vulva patterning and morphogenesis, found in the nematodes [88]. Genetic analyses of the different genes involved in fin development in zebrafish revealed the fibulin-6 and furin genes as potential Fraser syndrome disease (characterized by syndactyly and cryptophthalmos) genes [89]. The above study by Carney et al. (2010) showed that the loss of fibulin-6 (in the mutant zebrafish model) affected the attachment of the basement membrane to the underlying dermis, but the integrin-mediated cell attachment of epidermal cells to the basement membrane was not affected. In this way, cell–cell adhesion among epidermal cells was maintained, but the fin fold was lifted away from the underlying dermis as an intact epidermal sheet, and caused massive blistering (affecting the fin development). This study suggested the involvement of fibulin-6 in Fraser complex-dependent basement membrane anchorage, and the mutant zebrafish model as a suitable model to unravel the aetiology of human Fraser syndrome [89]. 

#### 3.3.2. Fibulin-8

The glycoprotein fibulin-8 (also known as hemicentin-2) has a molecular weight of 600 kDa and is expressed in developing somites and mesenchymal cells of zebrafish fins. It has been observed that fibulin-8 and fibulin-1 knockdown fish develop trunk blisters, which signify that both of these proteins play role in the migration of mesenchymal cells and the formation of the epidermal–dermal junction during development in zebrafish [90].

## 4. Role of Fibulins during Cancer Progression

Various studies reveal the involvement of fibulins in the pathogenesis of cancer diseases (Table 2). Understanding their role in cancer progression will pave the way for in-depth research on these proteins and promote the development of targeted therapeutics.

The glycoprotein fibulin-1 seems to possess both tumor suppressive and tumor enhancing effects, as revealed from various studies. There is a decreased expression of the fibulin-1D splice variant in the human fibrosarcoma tumor cell lines [92]. Moreover, this protein hinders the motility and adhesion of various types of cells, including melanoma, MDA-MB-231 breast carcinoma, and epidermal carcinoma cells [93]. On the other hand, an increased expression of fibulin-1 has been found in the sera of breast cancer patients, suggesting its role in the progression or pathogenesis of breast cancer [94]. Additionally, in patients with Philadelphia-negative chronic myeloproliferative neoplasms (MPNs), a significantly increased expression of fibulin-1 is observed, which in turn might enhance the capacity of TGF- β, as TGF- β anticipates various regulatory functions shown by fibulins and promotes cancer progression [95]. In contrast, the interaction of fibulin-1 with ADAMTS-1 in breast cancer cell lines induces an anticancer effect [96]. Another study on ovarian cancer reported an increased expression of the fibulin-1C splice variant, concluding that fibulin-1C promotes the progression of ovarian cancer [97]. Hence, fibulin-1 plays a dual role in promoting and suppressing the progression of the tumor, depending on the cell types; however, an in-depth study is needed to understand the mechanisms.

**Figure 2:**
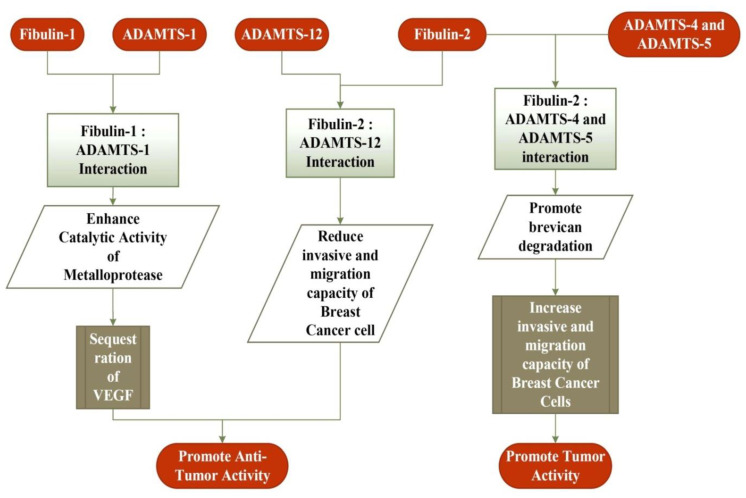
Interaction of fibulin-1 and fibulin-2 proteins with a disintegrin and metalloproteinase with thrombospondin motifs (ADAMTS) family of metalloproteases in breast cancer cells. The interaction of fibulin-1 with ADAMTS-1 promotes an anti-tumor activity by removing VEGF, a key mediator in cancer development. The interaction of fibulin-2 with ADAMTS-12 reduces the invasive and migration capacity of cancer cells, and thus promotes its anti-tumor activity. In contrast, fibulin-2 interaction with ADAMTS-4 and ADAMTS-5 promotes the tumor activity by increasing the invasive and migration capacity of cancer cells.

The identification of fibulin-2 as one of the 64 over-expressed metastasis-associated genes suggests its role in tumorigenesis [98]. Its expression is highly up-regulated in lung adenocarcinoma cell lines originating from mutant KP mice [99]. Additionally, the mutant KP cells express MUC4, which hinders the interaction of nidogen with fibulin-2, in turn splitting the integrity of the basement membrane and resulting in the spread of pancreatic cancer cells [100]. In human nasopharyngeal carcinoma (NPC), the expression of fibulin-2 is down-regulated, suggesting its tumor-suppressive and anti-angiogenic role [101]. In Kaposi’s sarcoma, the expression of fibulin-2 is decreased (along with the expression of fibulin-3 and -5), supporting the notion that a lower expression of fibulin-2 stimulates the wild proliferation, invasion, and migration of the cells [102]. The expression of the fibulin-2 protein was significantly more decreased in breast fibroadenoma tissues than for the normal tissues. Furthermore, its expression is down-regulated in more poorly differentiated tumor tissues than that of the differentiated tumor tissues for breast cancer, suggesting the influence of fibulin-2 in breast cancer proliferation and metastasis [103]. Furthermore, a decreased expression of fibulin-2 is observed in the breast cancer cell line, and the reintroduction of the same in the cell lines decrease the invasion and motility of cancer cells, suggesting the role of fibulin-2 in inhibiting the progression of cancer [104]. In breast cancer, the interaction between the fibulin-2 and ADAMTS-12 proteins promotes antitumor effects, but the absence of fibulin-2 evokes the pro-tumor effect of ADAMTS-12 in breast cancer cells (Figure 2) [105]. In contrast, fibulin-2 favors the malignant progression of lung adenocarcinoma by enhancing the attachment of tumor cells to collagen and collagen cross-linking [98]. Similarly, like fibulin-1, an increased expression of fibulin-2 has been reported in MPN patients [95]. Moreover, the interaction of fibulin-2 with ADAMTS-4 and ADAMTS-5 proteins favors the progression of breast cancer through the degradation of fibulin-2 (Figure 2) [106]. All of these findings suggest that fibulin-2 promotes and inhibits tumor progression, depending on the cell types, degree of malignancy, and stage of cancer. 

**Figure 3:**
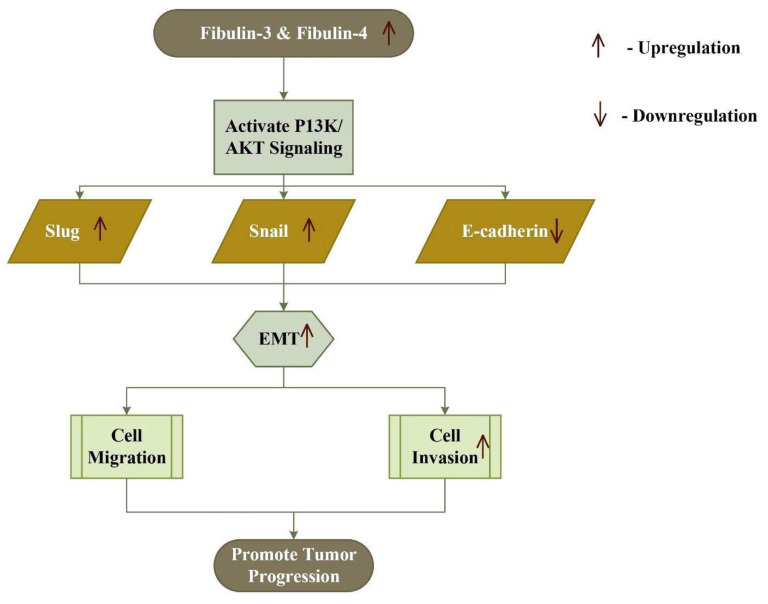
Increased expression of fibulin-3 and fibulin-4 activates the PI3K/AKT signaling pathway, which in turn up-regulates Snail and Slug, and down-regulates E-cadherin, thus inducing epithelial–mesenchymal transition (EMT) and tumor progression.

The expression of fibulin-3 is associated with an increased risk of glioma and breast cancer [107,108]. The effect of fibulin-3 depends on both the cell types and stage of development of cancer, like that of fibulin-2. Its expression is up-regulated in cervical cancer, osteosarcoma, glioma, and glioblastoma, whereas it’s down-regulated in prostate, colon, lung, liver, thyroid, breast, nasopharynx, and endometrial carcinoma [109,110,111,112,113,114,115,116,117,118,119,120,121]. In osteosarcoma cells, fibulin-3 promotes metastasis and invasion by activating the Wnt/β-catenin pathway, and promotes EMT by activating the PI3K/AKT pathway (Figure 3) [108]. In contrast, it exhibits inhibitory effects on EMT and a self-renewal capacity in the lung adenocarcinoma [122]. It is observed that a higher expression of fibulin-3 suppresses TGF-β-induced EMT, endothelial permeability, cell migration, and invasion in breast cancer cells [123]. Moreover, in advanced pancreatic adenocarcinoma, higher expression of fibulin-3 promotes angiogenesis, mediated by VEGF to enhance the cancer progression [124]. These findings suggest that the fibulin-3 exhibits both pro and anti-neoplastic effects as observed in fibulin-1 and -2.

Similarly, the glycoprotein fibulin-4 plays both pro-oncogenic and anti-oncogenic roles, as a higher expression of fibulin-4 leads to tumor progression in cervical, glioblastoma, and ovarian carcinoma, and a decreased expression leads to a poor prognosis of endometrial cancer [69]. In colon cancer, the expression of fibulin-4 mRNA increases significantly, favoring tumor progression [56]. In osteosarcoma, an increased expression of fibulin-4 favors metastasis and invasion, by inducing epidermal mesenchymal transition through the PI3K/AKT pathway and Wnt/β-catenin pathway (Figure 3) [125]. On the other hand, a higher expression of fibulin-4 in endometrial carcinoma inhibits cell invasion, proliferation, metastasis, and Wnt/β-catenin mediated epidermal mesenchymal transition [126].

**Figure 4:**
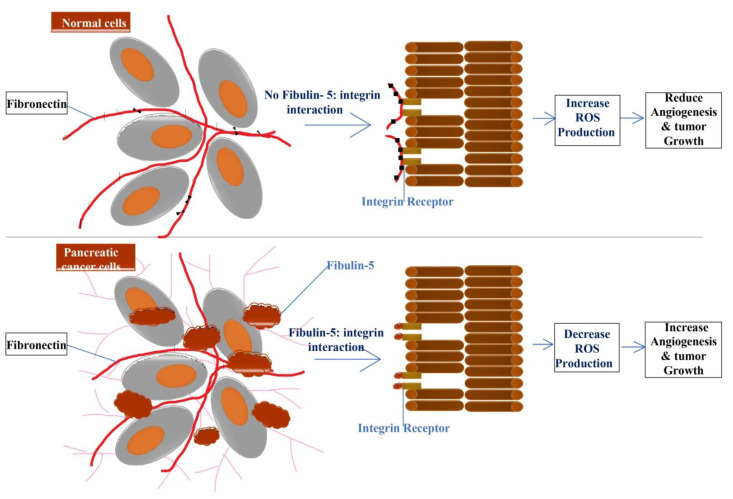
Fibulin-5 secreted by tumor-associated fibroblast blocks the interaction of fibronectin and integrin, which leads to a decrease or block of ROS (Reactive Oxygen Species) production resulting in increased angiogenesis and tumor growth. In contrast, in the absence of fibulin-5 and integrin interaction in normal cells, increased ROS production results in decreased angiogenesis and tumor growth.

Similar to the other types of fibulins, fibulin-5 may also promote or inhibit tumor progression. In fibrosarcoma, its higher expression promotes cell migration and tumor progression, whereas it’s decreased expression is observed in many human cancers, including breast, colon, kidney, and ovary cancer [15]. Moreover, in epithelial ovarian cancer (EOC) and human endometrial cancer, its expression is down-regulated, which signifies that fibulin-5 acts as a tumor suppresser for ovarian cancer [127,128,129]. Several hepatocellular carcinoma (HCC) cell lines display a low expression of fibulin-5, and suggest that fibulin-5 may inhibit HCC invasion and metastasis by suppressing the MMP-7 expression [130]. In a recent study on high-grade serous ovarian carcinoma (HGSOC), a lower expression of fibulin-5 was noted in the cancerous sample compared with that of the normal sample [131].In pancreatic ductal adenocarcinoma, fibulin-5 promotes tumor progression by blocking the reactive oxygen species production through competing with fibronectin for integrin binding sites, resulting in increased angiogenesis and tumor growth (Figure 4) [132].

A recent study on breast cancer revealed that the injection of the fibulin-7 protein in an animal model with breast tumors delayed the reprogramming of tumor-associated macrophages (TAMs) mediated by the negative regulation of the STAT3 pathway, which suggests that fibulin-7 may become a potential anti-cancer therapeutic agent [133]. Among the astrocytic tumors, fibulin-7 is highly expressed in glioblastoma tissues, including endothelial cells, pericytes of the glomeruloid, and hypertrophied microvessels [134]. An in-depth study is required to explore the involvement of fibulin-7 in cancer. 

## 5. Role of Fibulins in Other Diseases

An over- or under-expression of the fibulins may lead to different pathological conditions. A decreased expression of fibulin-1D leads to the development of heritable disorders such as autosomal-dominant giant platelet syndromes and congenital hand malformation [91]. Autosomal-dominant giant platelet syndromes (Fechtner, Sebastian platelet syndrome, Epstein, and May-Hegglin anomaly) display a broad spectrum of phenotypes, resulting from the different mutations in the nonmuscle myosin heavy chain 9 gene (MYH9). In this study of eight unrelated families with giant platelet syndromes, the mutation in the splice acceptor site of the fibulin-1D variant was observed in the absence of a MYH9 mutation, and was associated with an over-expression of antisense RNA [135]. On the other hand, congenital hand malformation patients display a synpolydactyly phenotype, which is caused by the reciprocal translocation between fibulin-1 gene (located on 22q13.3 chromosome) and C12orf2 segment (on the short arm of the 12th chromosome) [136].

Furthermore, the Arg345Trp single mutation in the fibulin-3 (*FIBL3*) gene is associated with Malattia Leventinese and Doyne honeycomb retinal dystrophy, an autosomal dominant retinal disease [47]. Malattia Leventinese and Doyne honeycomb retinal dystrophy are macular degenerative disorders characterized by the appearance of small round yellow-white spots in the macula of the retina (between retinal pigment epithelium and Bruch’s membrane), which form a honeycomb pattern during early adulthood [137].

Mutations in the fibulin-4 gene cause many molecular defects, such as those affecting the rate of secretion, stability of the protein molecules of the TGF-β pathway, and cross-linking process [138]. The cutis laxa form of the autosomal recessive disorder is one of the defects caused by a E57K missense mutation in the fibulin-4 gene [139]. Compound heterozygous and homozygous mutations in the fibulin-4 gene cause arterial tortuosity, diaphragmatic and inguinal hernia, ascending aortic aneurysms, developmental emphysema, joint laxity, arachnodactyly, and bone fractures with different severities [69]. Fibulin-4 knock-in mice with an E57K missense mutation display vascular, skeletal, and pulmonary abnormalities [140].

The Ser227Pro homozygous missense mutation in the fibulin-5 gene results in a severe autosomal recessive form of cutis laxa [141]. Furthermore, the dysregulation of fibulin-6 has been associated with the salivary gland autoimmune diseases Sjogren’s syndrome [142] and Fraser syndrome, as it has a potential role in Fraser complex-dependent basement membrane anchorage [89].

## 6. Miscellaneous Role of Fibulin Family 

Besides their involvement in embryonic stage development and diseases, fibulins also have some other physiological functions, which are discussed here. Fibulin-1D and fibulin-2 interact with the sex hormone-binding globulin and regulate the action of steroid hormones in the ECM [143]. Moreover, the expression of fibulin-1 has been noticed in the glandular epithelium in the proliferative phase endometria, and this expression pattern is switched to the stromal cells in the secretory phase endometria [27]. The in vitro (human endometrial stromal cells) and in vivo (human endometrial tissue) findings reveal that progesterone induces fibulin-1 expression in the stroma of the human endometrium during the decidualization (stromal cell differentiation) process. These findings suggest that fibulin-1 might play an important role in human endometrial stromal cell differentiation [27]. Moreover, during interpubic ligament development, the expression of fibulin-5 is regulated, suggesting its role in the formation of new elastic fibers [144]. In bovines, the expression of the fibulin-2 precursor is found to increase during early pregnancy [145]. Additionally, in pregnant cow urine, an increased expression of the fibulin-2 X1 isoform has been reported, suggesting that fibulin-2 may act as a biomarker for early pregnancy diagnosis in cattle [146].

## 7. Conclusions

Fibulins are the crucial glycoproteins present in the ECM, with various structural and physiological functions. These proteins play a vital role in different developmental stages of the embryo and, as research findings have revealed, they are involved in both tumor-suppressing and tumor-promoting activities, depending on the cell types and the tissue microenvironment. The expression of different members of the fibulin family varies at different stages of development. Fibulins act as intermolecular bridges and mediators to form supramolecular structures, promote tissue remodeling, and execute cellular processes. Knockout models demonstrate the role of fibulins in various developmental and pathological conditions, including elastogenesis, vascularization, and cancer development. However, in-depth studies are required to understand the dual role of fibulins, such as a cancer-suppressive and cancer-promoting activity in different microenvironmental milieu in humans. Targeted therapeutics can be developed in the future, with fibulins as molecular targets in various cancer diseases, after a thorough understanding of their role in cancer progression at a molecular level. Likewise, as the literature describes the role of fibulin in embryonic development, future research can emphasize establishing fibulin as a biomarker for early pregnancy detection in farm animals.

## Figures and Tables

**Table 1:** Size, chromosome location, and expression pattern of fibulins.

S.No.	Types of Fibulins	Size (kDa)	Gene Location	Location in ECM	Expression Pattern	References
1.	Fibulin-1	90-100	22q13.31	Fibril matrix	Cartilage, developing limbs, developing myotomes, perichondrial structures, neural crest cells, and endocardial cushion tissue	[7,26,45]
2.	Fibulin-2	~195	3p24-p25	Basement membrane	Developing heart, smooth muscle precursor cells, developing cartilages, neural crest cells, and endocardial cushion tissue	[40,42]
3.	Fibulin-3	~50	2p16	Basement membrane	Cartilage, developing bones, and developing cranial area	[50]
4.	Fibulin-4	~50	11q13	The interface between the fibrillin microfibrillar scaffold and the elastin core	Heart, skeletal muscle, placenta, lungs, pancreas, brain, and kidney	[2]
5.	Fibulin-5	~65	14q32.1	Basement membrane	Developing artery, neural crest cells, mesenchymal tissues, endothelial cushion tissue, heart, lungs, and uterus	[39,65,66,91]
6.	Fibulin-6	~600	1q25.3	Basement membrane	Retinal endothelial and epithelial cells, and skin fibroblasts	[3,39,86]
7.	Fibulin-7	~48	2q13	Pericellular region	Cartilage, placenta, teeth, and hair follicles	[10]
8.	Fibulin-8	~600	9q34.11	Basement membrane	Developing somites and mesenchymal cells in zebrafish	[39,90]

**Table 2:** Fibulins in different types of cancer and other diseases.

Type of Fibulin	Type of Cancer	Role	Involvement in Other Diseases	References
Fibulin-1	Breast cancer	Increased expression promotes cancer progression	Autosomal dominant giant platelet syndrome and congenital hand malformation	[91,94,95,96,97]
	Ovarian cancer	Increased expression promotes cancer progression		
	Interaction with ADAMTS-1 in breast cancer	Promotes anti-tumor effect		
	Philadelphia-negative chronic myeloproliferative neoplasms (MPNs)	Increased expression enhances the activity of TGF-β and promotes cancer progression		
Fibulin-2	Human nasopharyngeal carcinoma	Tumor suppressive and anti-angiogenic effect		[99,101,102,103,105,106]
	Kaposi’s sarcoma	Lower expression stimulates wild proliferation, invasion, and migration of the cancer cells		
	Breast fibroadenoma	Decreased expression promotes cancer progression		
	Interaction with ADAMTS-12 in breast cancer	Promotes anti-tumor effects		
	Lung adenocarcinoma	Favors malignant progression of lung cancer		
	Interaction with ADAMTS-4 and ADAMTS-5 in breastcancer	Favors cancer progression		
Fibulin-3	Osteosarcoma	Promotes metastasis and invasion by activating Wnt/β-catenin pathway and EMT by PI3K/AKT pathway	Malattia Levantines and Doyne honeycomb retinal dystrophy	[51,108,122,123,124]
	Lung adenocarcinoma	Exhibits inhibitory effects on EMT and a self-renewal capacity		
	Breast cancer	Suppresses TGF-β induced EMT and a self-renewal capacity		
	Pancreatic adenocarcinoma	Promotes cancer progression		
Fibulin-4	Colon cancer Cervical cancer Glioblastoma Ovarian carcinoma	Increased mRNA expression favors tumor progression	Cutis laxa, arterial tortuosity, diaphragmatic and inguinal hernia, ascending aortic aneurysms, developmental emphysema, joint laxity, and arachnodactyly	[56,69,125,126,139]
	Osteosarcoma	Increased expression favors metastasis and invasion		
	Endometrial carcinoma	Higher expression inhibits cell invasion, proliferation, metastasis, and Wnt/β-catenin mediated EMT		
Fibulin-5	Fibrosarcoma	Higher expression promotes cell migration and tumor progression	Ser227Pro homozygous missense mutation causes cutis laxa	[15,16,17,18,19,20,21,22,23,24,25,26,27,28,29,30,31,32,33,34,35,36,37,38,39,40,41,42,43,44,45,46,47,48,49,50,51,52,53,54,55,56,57,58,59,60,61,62,63,64,65,66,67,68,69,70,71,72,73,74,75,76,77,78,79,80,81,82,83,84,85,86,87,88,89,90,91,92,93,94,95,96,97,98,99,100,101,102,103,104,105,106,107,108,109,110,111,112,113,114,115,116,117,118,119,120,121,122,123,124,125,126,127,128,129,132,141]
	Epithelial ovarian cancer and human endometrial cancer	Acts as a tumor suppressor		
	Pancreatic ductal adenocarcinoma	Promotes tumor progression by blocking reactive oxygen species production		
Fibulin-6	-	-	Sjogren’s syndrome and Fraser syndrome	[89,142]
Fibulin-7	Breast cancer	Delays reprogramming of the tumor associated macrophages		[132,133]
	Astrocytic tumor	Promotes tumor progression		
